# Design of a data processing method for the farmland environmental monitoring based on improved Spark components

**DOI:** 10.3389/fdata.2023.1282352

**Published:** 2023-11-20

**Authors:** Ruipeng Tang, Narendra Kumar Aridas, Mohamad Sofian Abu Talip

**Affiliations:** Department of Electrical Engineering, Faculty of Engineering, University of Malaya, Kuala Lumpur, Malaysia

**Keywords:** SPARK, FAST-join algorithm, agricultural big data, agricultural cloud technology, agricultural data processing

## Abstract

With the popularization of big data technology, agricultural data processing systems have become more intelligent. In this study, a data processing method for farmland environmental monitoring based on improved Spark components is designed. It introduces the FAST-Join (Join critical filtering sampling partition optimization) algorithm in the Spark component for equivalence association query optimization to improve the operating efficiency of the Spark component and cluster. The experimental results show that the amount of data written and read in Shuffle by Spark optimized by the FAST-join algorithm only accounts for 0.958 and 1.384% of the original data volume on average, and the calculation speed is 202.11% faster than the original. The average data processing time and occupied memory size of the Spark cluster are reduced by 128.22 and 76.75% compared with the originals. It also compared the cluster performance of the FAST-join and Equi-join algorithms. The Spark cluster optimized by the FAST-join algorithm reduced the processing time and occupied memory size by an average of 68.74 and 37.80% compared with the Equi-join algorithm, which shows that the FAST-join algorithm can effectively improve the efficiency of inter-data table querying and cluster computing.

## 1 Introduction

In the application of the Internet of things, technology in agricultural greenhouses, sensors, Zigbee, cameras, and other devices are widely used. For real-time monitoring and requirements, these devices will set the collection frequency very high, resulting in a rapid increase in the amount of collected data, and these data also need to be calculated and analyzed, which requires high timeliness of data processing. Traditional agricultural data processing techniques are increasingly unable to meet the technical requirements. The integration and extraction of agricultural big data based on the Internet of things require big data technology support (Tao et al., 2020; Mourtzis et al., 2021). Therefore, one of the current agricultural big data research directions is how to quickly process and analyze the massive redundant and low-value farmland environmental monitoring data.

To this end, some scholars have researched the application of agricultural big data and achieved good results (Cheng and Zhang, 2019). Lamrhari et al. (2016) processed and analyzed the collected environmental data to build an effective agricultural big data architecture to assist manufacturers and consulting companies make decisions, improve agricultural productivity and monitoring capabilities, and achieve better results. Dean and Ghemawat (2004), aiming at the data status of the agricultural Internet of things, proposed a cloud mobile architecture based on distributed storage based on data dispersion and discontinuity characteristics. Gabriel (2016), based on the long-term and dynamic monitoring of soil information, etc., built a soil model database based on the above data and applied it in actual production. Xiangbao et al. (2014) proposed a SMART agricultural big data system framework in 2014, expounded on the leading technologies, related resources, and main application scenarios of agricultural big data, and developed an agricultural big data platform based on intelligent analysis. Xiufeng et al. (2014) proposed to establish a visual interactive system in the interactive technology of agricultural big data services, which provides users with multi-level network data services. Wensheng and Leifeng (2015) and Leifeng (2016) summarized the big data available in each period according to the characteristics of agricultural development and provided detailed guidance on the acquisition of data sources, complex environmental factors in farmland, crop agronomic parameters, etc. The pre-arrangement provided technical method support. Yang et al. (2011) proposed a Hadoop-based large file block storage method and a massive agricultural data resource retrieval method, which support realizing the efficient organization and management of massive agricultural data resources.

However, many scholars focus on the application and model construction of big data in agriculture and rarely address the massive data processing methods and processes of big data in agriculture. In addition, as most of the research is based on the Hadoop ecosystem, efficient Spark Structured Query Language (SQL) and other related mechanisms have not been introduced, and the processing efficiency needs to be improved. Apache Spark is an open-source, distributed, high-performance, and massive data processing engine (Weihua et al., 2020). Although Flink performs better in real-time computing, its development history is far shorter than Spark. Its integration with Hadoop ecosystem components is far inferior to Spark, and Spark also supports offline computing and offline computing tasks in big data queries. The running proportion of offline computing tasks is much higher than that of real-time computing tasks. At the same time, Spark also supports machine learning modeling, and its support for algorithm models is much stronger than that of Flink. Therefore, this study proposes a data processing method for farmland environmental monitoring based on improved Spark components. The Spark component is optimized and applied to the data processing process of the farmland environmental monitoring to perform rapid calculation and analysis on farmland environmental monitoring data with massive redundancy and low data value density.

## 2 Spark optimization method design

### 2.1 Spark component optimization

In Spark computing, the most important function is tables' equivalent join and statistical operation. The core of the table association operation is the Join Key operation (Jun et al., 2015), which is usually partitioned according to the Join Key, and all records with the same key value are equivalently connected in each partition. Table equivalence join operations are many, and the problem of Join Key data skew is prone to occur, which will produce Shuffle. It will move data among nodes in different clusters, which is a time-consuming and resource-consuming operation, affecting the efficiency of data queries.

To this end, this study introduces the FAST-Join (Join Key filtering sampling partition optimization) algorithm. Through the generated BloomFilter filter (Khan et al., 2022), the data tables to be joined are sorted and deduplicated in advance, and the data that does not meet the connection conditions are filtered out to reduce the amount of data in the Shuffle stage, thereby reducing network transmission volume and disk read and write overhead and optimizing the execution efficiency of Join operations. The FAST-Join algorithm proposed in this study mainly has three parts: Connection property handling, Join Key sampling, and Data table splitting.

### 2.2 Connection property handling

**Figure 1:**
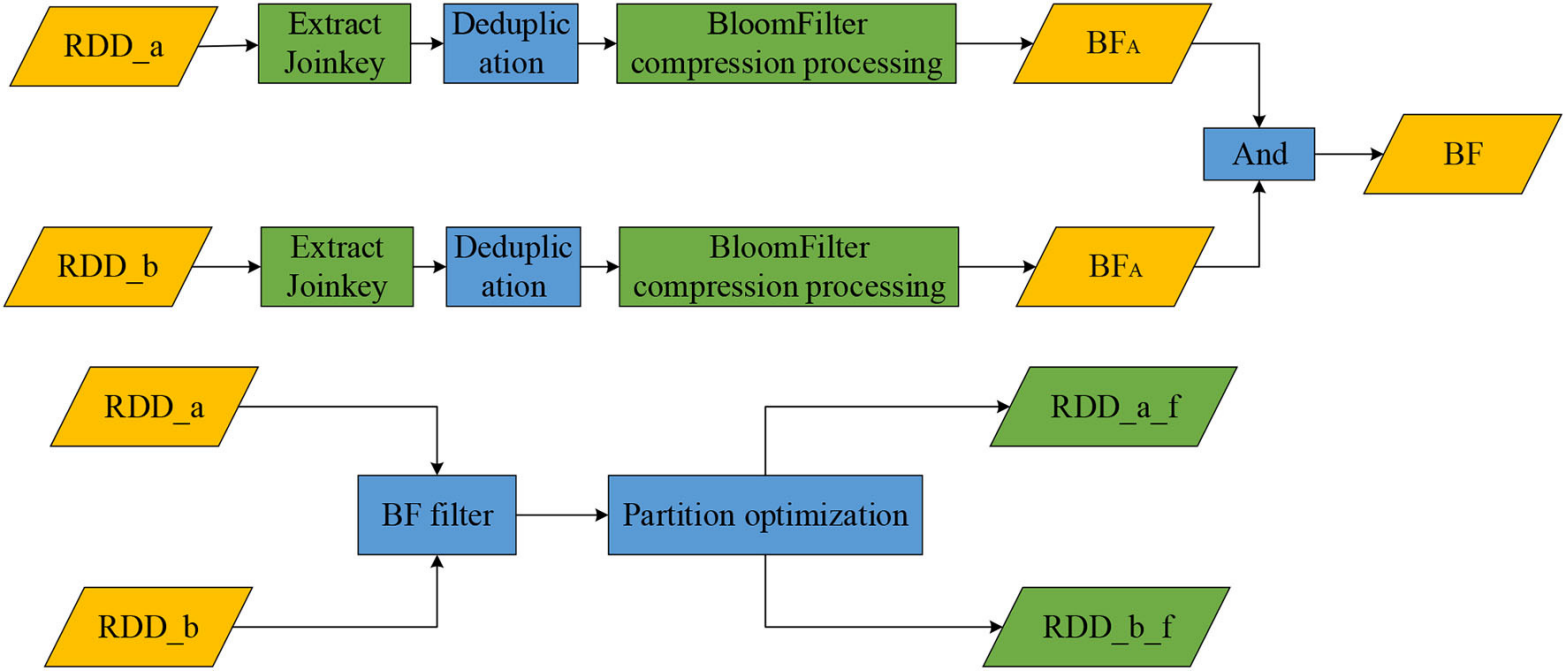
The connection property handling process.

This algorithm extracts two data tables, RDD_a and RDD_b, which are extracted to get the Join Key after the Join operation. Then it obtains two new RDDs (Join Key_a and Join Key_b) and obtains the duplicate set of Join Key_a and Join Key_b through the deduplication operation. It uses the BloomFilter of the Spark class library to analyze the data. The data sets of Join Key_a and Join Key_b are calculated to obtain two bit arrays, BFA and BFB. Finally, the And operation is performed on BFA and BFB to generate the final filtered bit array, BF. Figure 1 shows the connection property handling process. Table 1 show the symbols of the algorithms.

**Table 1:** The symbols of the algorithms.

**Name**	**Value**
RDD_a	Data table a
RDD_b	Data table b
Join Key_a	Connection properties of RDD_a
Join Key_b	Connection properties of RDD_b
BFA	BloomFilter bit array of RDD_a
BFB	BloomFilter bit array of RDD_b
BF	BloomFilter bit array
RDD_a_f	Partitioned RDD_a
RDD_b_f	Partitioned RDD_b
*RDD*_*ai*_	RDD_a split partition
*RDD*_*bi*_	RDD_b split partition
*T*_*ai*_	Data volume of *RDD*_*ai*_ estimation quantization value
*T*_*bi*_	Data volume of *RDD*_*bi*_ estimation quantization value
*T*	Data volume estimation quantization value array
*OS*_*k*_	Join Key corresponding data volume estimate
*OS*	Broadcastable data amount quantification threshold

### 2.3 Join Key sampling

**Figure 2:**
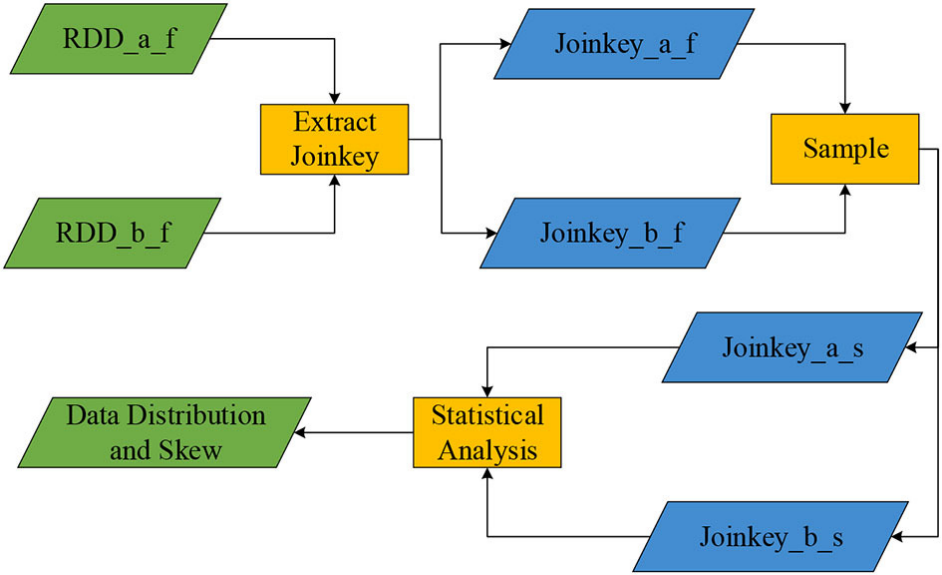
The Join Key sampling process.

This algorithm extracts Join Key operations on the two filtered partitions RDD_a_f and RDD_b_f, which can obtain two sets of Join Key_a_f and Join Key_b_f (Song et al., 2018). It uses the sample operator on the two RDDs of Join Key_a_f and Join Key_b_f to sample the Join Keys of the two RDDs to obtain Join Key_a_s and Join Key_b_s (the sampling rate is set to 0.2). The statistical methods of the Spark Statistics library are used to analyze and compare the two sample sets. Finally, it gets the skew of data in the single RDD, the total amount of data corresponding to the Key in the single table, and the data distribution of the same Key value in two partitions. Figure 2 shows the Join Key sampling process.

### 2.4 Data table split

Split the two data tables according to sampling analysis results, divide them according to the distribution and inclination of the data of the connection attributes, and use the principle of converting reduced side join into map join as much as possible and reduce the data volume of Shuffle operation aggregation (Chunhui, 2015), which associates the degree of inclination with the distribution. The splitting criteria are as follows:

- The number of RDD_a split partitions cannot exceed the configured number of splits;
- For Keys with serious data skew in RDD, split them out directly;
- After splitting out the Key with serious data skew in the RDD, the split partition starts with the smaller Key in the sample that is sampled in a single RDD. The *OS*_*k*_ of the Key superposes *OS*_*k*_, which is compared with OS every superposition. If it is bigger than the OS, the split mark is the previous Key, and the superposition variable is reset to the *OS*_*k*_ of the current Key.
- Split the sampled key separately, and split it forward according to the split mark in the number of remaining splits.

**Figure 3:**
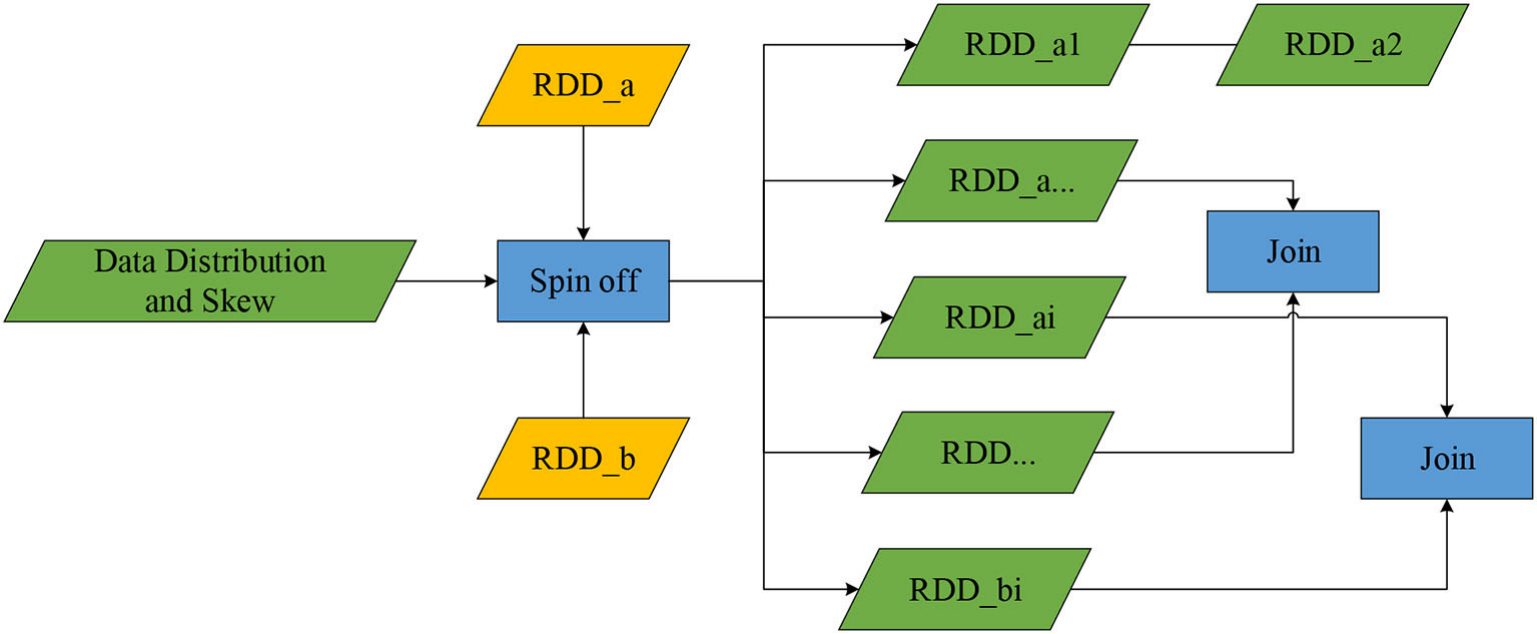
The *data table split* process.

According to the estimated value Ti and T of the aggregated data table *RDD*_*ai*_ scale, the splitting mentioned above action will be completed. According to the judgment result, if *RDD*_*ai*_is small-scale data, then perform BroadcastJoin on *RDD*_*ai*_ and its corresponding *RDD*_*bi*_, otherwise perform Hash (Roller and Sukhbaatar, 2021) or Sort MergeJoin (Papaphilippou and Pirk, 2019); T is the threshold of data size, and there are three values corresponding to small, medium, and large scale. According to the above judging rules, join *RDD*_*ai*_and *RDD*_*bi*_ sequentially until all split partition joins are completed, and perform Union on all results. Finally, the data is processed by Spark and stored in the database Mysql for user query and visual display. Figure 3 shows the data table split process.

## 3 Experimental design and results

### 3.1 Research object introduction

**Figure 4:**
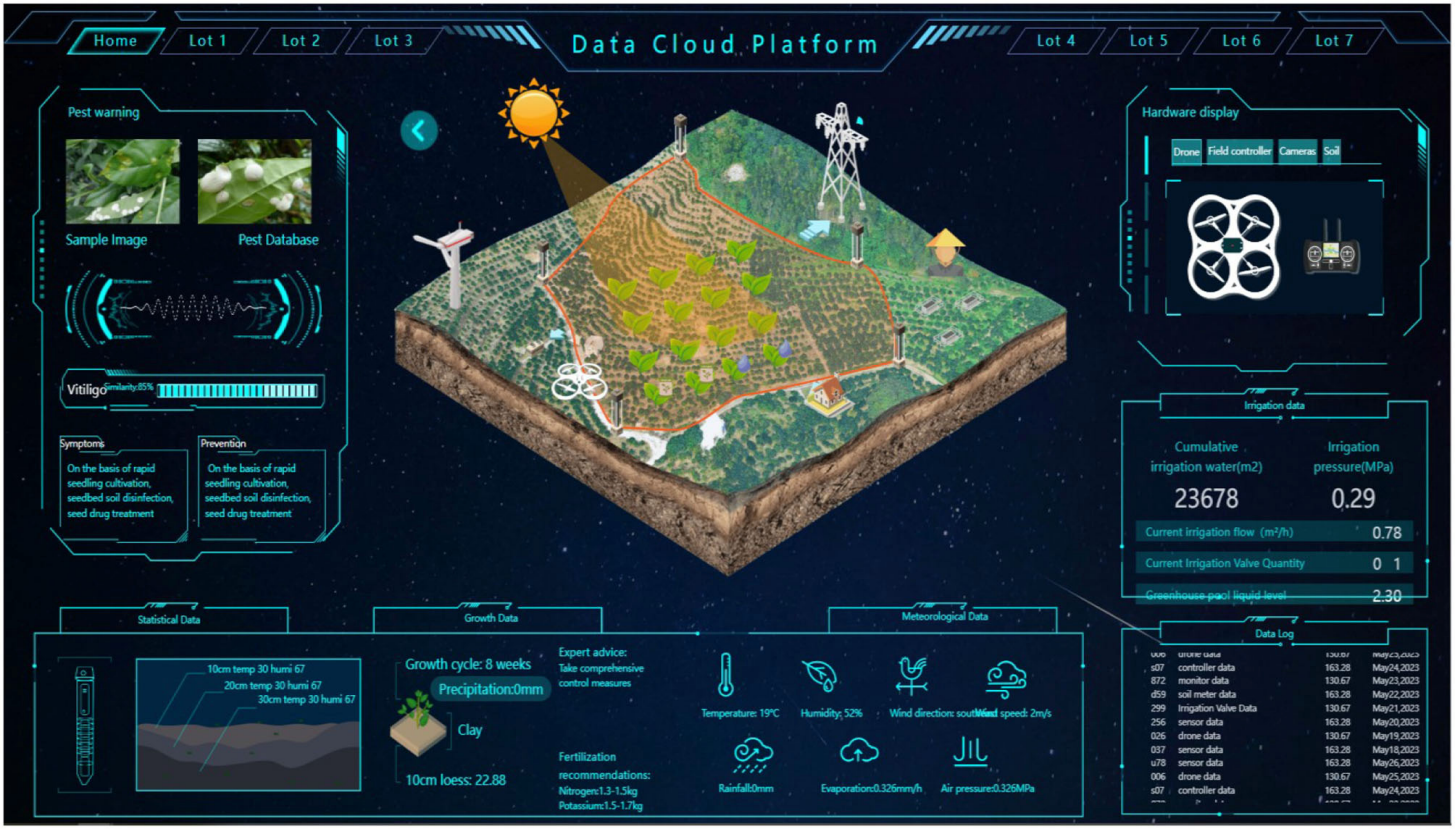
The homepage of the environmental monitoring cloud platform.

This study takes the data cloud platform of a farm in Malaysia as the experimental object. The system comprises the farmland environmental monitoring platform and management functions such as roles, plots, sensors, operations, and agricultural information. It can conduct comprehensive statistical analysis on all environmental monitoring-related information involved in the system. It manages 40 rice planting plots at the farm. Each plot has two high-definition cameras, four sets of sensor equipment for environmental monitoring, and one intelligent irrigation pump. The sensor equipment monitors the temperature, humidity, and wind direction of the plot, and wind speed, rainfall, evaporation, air pressure, nitrogen fertilizer content, potassium fertilizer content, sampling soil depth, soil type, and other parameters, and transmits data to the cloud platform through a Zigbee wireless sensor network (Chakraborty, 2021) and network router every minute. The log data is consumed by Kafka and transmitted to the Spark cluster for processing, and the data is displayed on the cloud platform after data processing and statistical summary. Its underlying component is Spark (Independent mode deployment) and other supporting components (Flume, Kafka, Mysql, Grafana, etc.) (Jose, 2020; Kaicheng et al., 2020; Xiaoxian et al., 2020). Figure 4 shows the homepage of the environmental monitoring cloud platform.

### 3.2 Experimental cluster design

This study's farmland environmental monitoring platform is carried out on the Aliyun server. Each server uses the environment of CentOS 7, running independently (Table 2 shows the server's configuration parameters). There are a total of 20 nodes. These nodes are divided into groups A and B, each with 10 nodes; Group B deploys the improved Spark components of the FAST-join algorithm (Spark table equivalence connection processing optimization). Group A deploys the default Spark component (Zidong and Yanbin, 2019), which is the large-scale RDF data based on SPARK. Finally, it compares the component performance and cluster performance.

**Table 2:** The configuration parameters of the server.

**Name**	**Value**
Number of CPU cores	2
Memory	2 G
Hard disk	50 G
Network	50 Mbps
Operating system	CentOS 7.4
Java version	1.8.4
Spark	2.1.1
Grafana	8.4.0
Kafka	2.12.1
HBase	2.5.0
Mysql	7.5.0

### 3.3 Experimental data

The farmland environmental monitoring platform involves a lot of data, which have mainly three aspects, such as the image recognition of diseases and insect pests, farmland environmental monitoring, and operating equipment feedback, and the forms of each type of data are quite different. The statistical time range is from 1 June 2022 to 30 May 2023, a whole year, and the data range is temperature, humidity, wind direction, wind speed, rainfall, evaporation, air pressure, nitrogen fertilizer potassium content, potash content, sampling soil depth, soil type, and other indicators. The average data of each period of each day are counted daily (farmland environmental monitoring data are sent to the data processing system by polling every minute). Table 3 shows the daily average of the environmental monitoring data of a certain plot at the farm for the first 12 days of May 2023.

**Table 3:** The daily average data of a certain plot at the farm for the first 12 days of May 2023.

**Date**	**Temperature (°C)**	**Humidity (%rh)**	**Wind direction**	**Wind speed (m/s)**	**Rainfall (mm)**	**Evaporation (mm)**	**Barometric pressure (Mpa)**	**Nitrogen (Kg)**	**Potassium (Kg)**	**Soil depth (cm)**	**Soil type**
2023.05.01	33.0	80.48	Southeast	5.55	6.02	4.32	108.45	143.25	40.70	19.06	Clay
2023.05.02	28.4	87.13	South	4.70	8.12	4.02	109.00	149.65	48.83	20.85	Clay
2023.05.03	30.8	85.73	Northwest	9.33	8.20	4.36	108.37	125.95	40.17	20.85	Clay
2023.05.04	31.2	84.34	Northeast	6.76	7.18	4.60	105.55	140.01	40.89	19.45	Clay
2023.05.05	30.8	86.66	East	6.53	7.78	4.96	108.58	132.07	44.09	20.74	Clay
2023.05.06	31.5	87.94	Southwest	5.30	6.73	4.60	105.42	140.83	49.48	19.42	Clay
2023.05.07	31.6	82.30	West	8.10	5.75	4.62	107.82	115.22	39.68	20.31	Clay
2023.05.08	30.3	86.75	Southeast	6.02	6.48	4.64	105.81	148.46	51.98	20.47	Clay
2023.05.09	29.8	83.01	North	4.90	6.54	4.41	107.84	147.29	35.24	20.91	Clay
2023.05.10	32.6	84.03	Southeast	4.96	5.17	4.18	105.69	121.65	51.29	19.74	Clay
2023.05.11	32.5	87.70	Northeast	8.67	7.85	4.38	106.95	151.47	40.47	19.87	Clay
2023.05.12	32.5	82.76	Northeast	8.67	6.84	5.00	106.40	132.99	49.08	20.27	Clay

### 3.4 Experimental result

#### 3.4.1 Spark component performance

In the statistical analysis of Spark components, most scenarios are equivalent joins of large tables, the core of which is the Join Key operation (Zidong and Yanbin, 2019). Shuffle will be generated during the Join process, and Shuffle will cause data skew during the Spark calculation process. The operating capability and resource consumption of the cluster and the processing of Shuffle are important indicators to measure the performance of Spark components. In this study, the default Spark cluster and improved Spark cluster are designed to deal with shuffle experiments. In order to compare the component performance of the two groups of clusters, A and B, this study exported part of the original data (the farmland environmental monitoring data) as a text file and copied it into two files (Data_A and Data_B). Data_A was used as a set of data sets, and Data_B was written with specific and skewed data as another set of size, so the different data sets have the different data skew. The two data sets to be joined in each group were RDD_a and RDD_b (Zhichao et al., 2022). Table 4 shows the simulation parameters.

**Table 4:** The simulation parameters.

**Data set**	**Data_A**	**Data_B**
RDD_a	4.7 G	5.4 G
RDD_b	5.1 G	6.7 G

In this experiment, Group A (the default Spark component) and Group B (Spark component improved by the FAST-join algorithm) were used to test the above two sets of data sets, and two query statistics experiments were carried out and compared separately. These two groups of Spark clusters perform the Join Key operation on the data set 1/2, which generates Shuffle, and the size of the Shuffle directly affects the cluster computing time and resource consumption. The smaller the Shuffle, the shorter the cluster computing time and the resource consumption. The degree of consumption is denpended on the computing time and resource consumption of the cluster. So the experimental results take the Shuffle read, write, and running time as comparison indicators (Singh et al., 2020), and each group of experiments is carried out three times, and the results are averaged. The experimental results are shown in Tables 5, 6.

**Table 5:** Shuffle write data volume.

**Cluster name**	**Data_A**	**Data_B**
Group A	264.8 M	356.1 M
Group B	2.53 M	3.37 M

**Table 6:** Shuffle read data volume.

**Cluster name**	**Data_A**	**Data_B**
Group A	231.9 M	314.2 M
Group B	3.17 M	4.4 M

Tables 6, 7 shows that the amount of data written and read by Shuffle in the two connection methods in the two groups of experiments changes significantly. Group B has much less than the data volume of Group A, whether read or written in Shuffle. The data volume of Group B accounts for only 0.958% of the Shuffle write and 1.384% of the Shuffle read data volume of Group A on average. The main reason is that the FAST-join algorithm performs BloomFilter filtering on unsatisfied connection conditions and redundant data before the connection, which reduces the overall Shuffle data volume during the connection. Table 7 shows the Runtime comparison of groups A and B under different data sets.

**Table 7:** Runtime comparison of groups A and B under different data sets.

**Cluster name**	**Data_A**	**Data_B**
Group A	48.2 min	63.9 min
Group B	15.7 min	21.5 min

**Figure 5:**
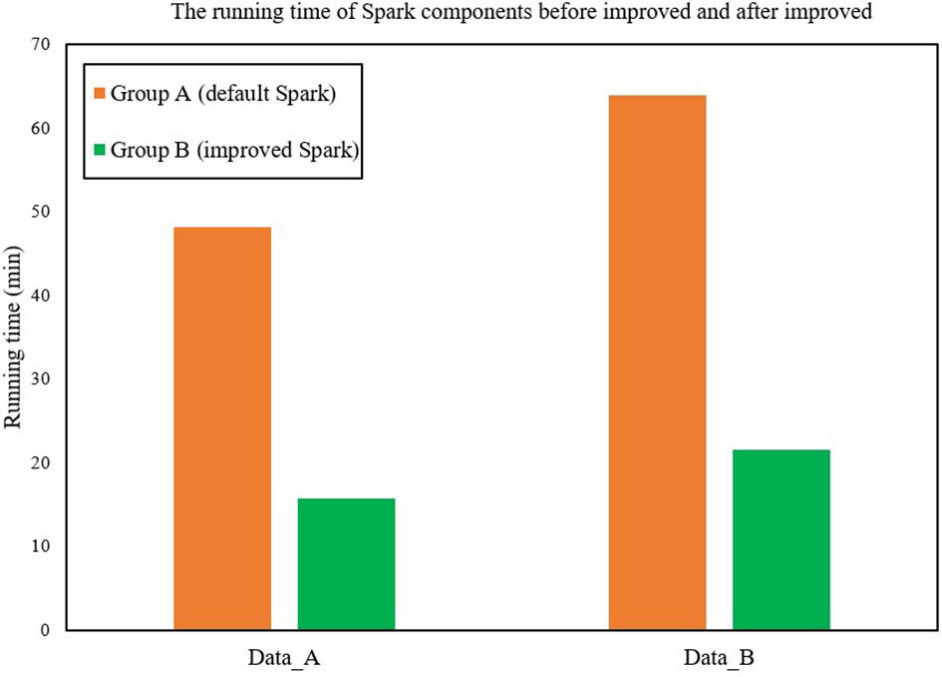
The running time of Spark components before and after improvement.

Figure 5 shows the running time of Spark components before and after improvement. For each group of experiments, the running time of Group B is significantly better than Group A (Raj and Ramesh, 2021). The average running time of Group B is only 33.11% of Group A's, and the calculation speed is 202.11% faster than that of Group A. The execution time of the FAST-Join algorithm is relatively smooth, while the two execution times of Spark's Join operation show a relatively large increase. The data skew is more serious.

#### 3.4.2 Spark cluster performance

Processing time and occupied memory size are important indicators to measure the overall performance of the Spark cluster, so this study used data of different magnitudes to test Group A and Group B and obtain the corresponding processing time and occupied memory size. In this experiment, 600,000 pieces of data were randomly selected, and three sets of data sets were generated, 100,000, 200,000, and 300,000 pieces, respectively.

**Figure 6:**
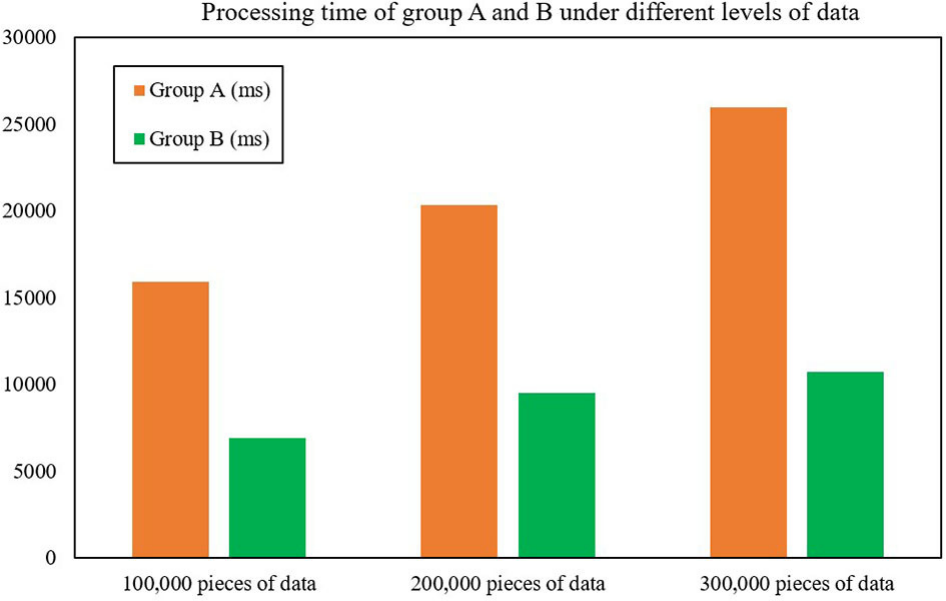
Processing time of groups A and B under different levels of data.

The first test was for processing time, which compares the processing time of Group A (the cluster composed of improved Spark components based on FAST-join algorithm) and Group B (the cluster composed of default Spark components). After experimental testing, the average data processing time of Group B was only 43.93% of that of Group A, an average reduction of 128.22% compared with Group A (Table 8 shows the validity test experimental data), which shows that the data processing efficiency of Spark optimized by the FAST-join algorithm is greatly improved compared with unoptimized Spark. Figure 6 and Table 8 show the processing time of groups A and B under different levels of data.

**Table 8:** Processing time of groups A and B under different levels of data.

**Data volume (100,000 pieces)**	**Group A (ms)**	**Group B (ms)**
1	15,907	6,945
2	20,376	9,528
3	25,958	10,737

**Figure 7:**
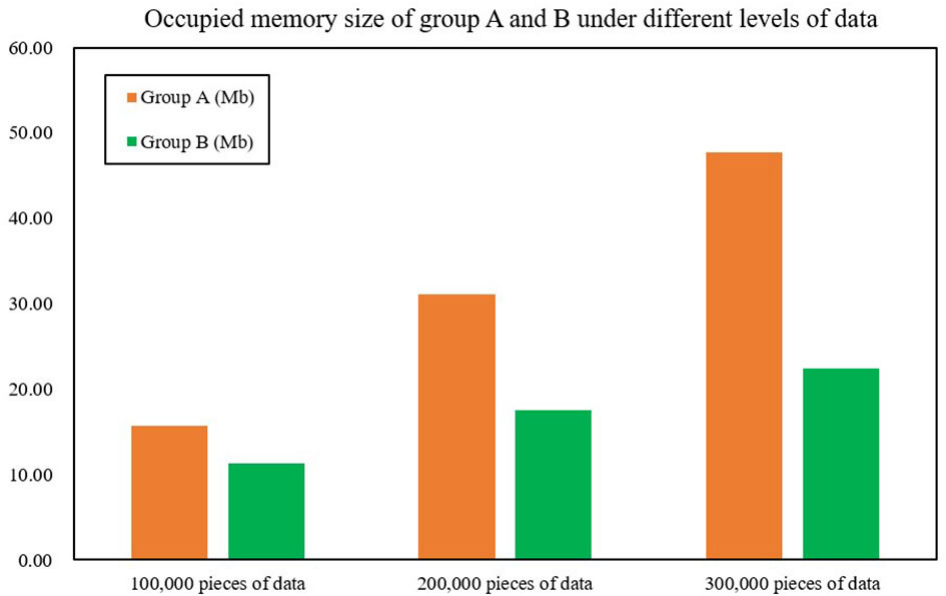
Occupied memory size of groups A and B under different levels of data.

The second test was for occupied memory size; the test and the processing time test are of the same proportion of data magnitude (100,000, 200,000, and 300,000 pieces). After monitoring by using Spark's built-in monitoring tool, the average data processing time of Group B only accounts for 58.28% of Group A's, an average reduction of 76.75% compared with Group A, which shows that the occupied memory size of Spark optimized by the FAST-join algorithm is significantly reduced compared with unoptimized Spark. Figure 7 and Table 9 show the occupied memory size of groups A and B under different levels of data.

**Table 9:** The occupied memory size of groups A and B under different levels of data.

**Data volume (100,000 pieces)**	**Group A (Mb)**	**Group B (Mb)**
1	15.70	11.25
2	31.12	17.48
3	47.74	22.46

### 3.5 Experimental extension

**Figure 8:**
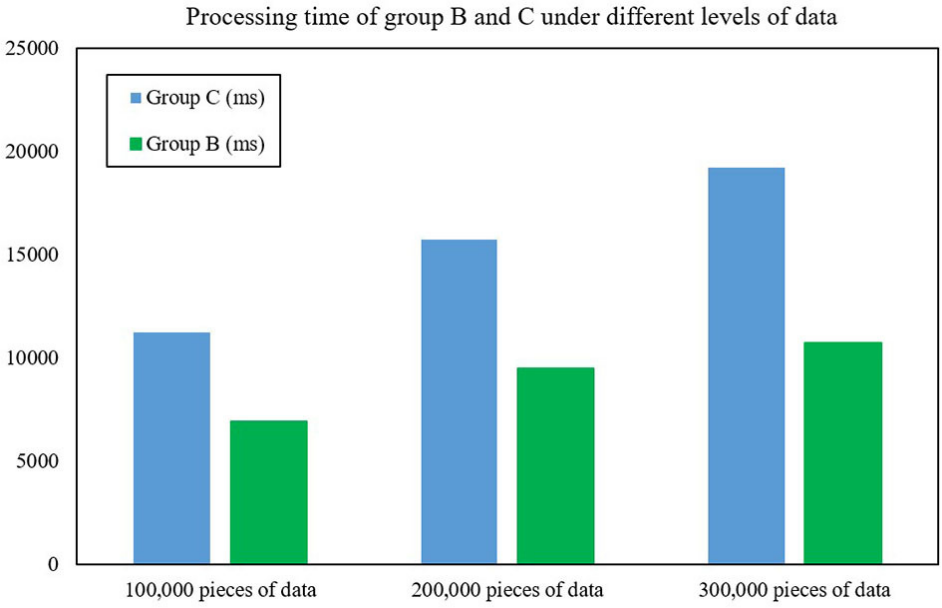
Processing time of groups B and C under different levels of data.

**Figure 9:**
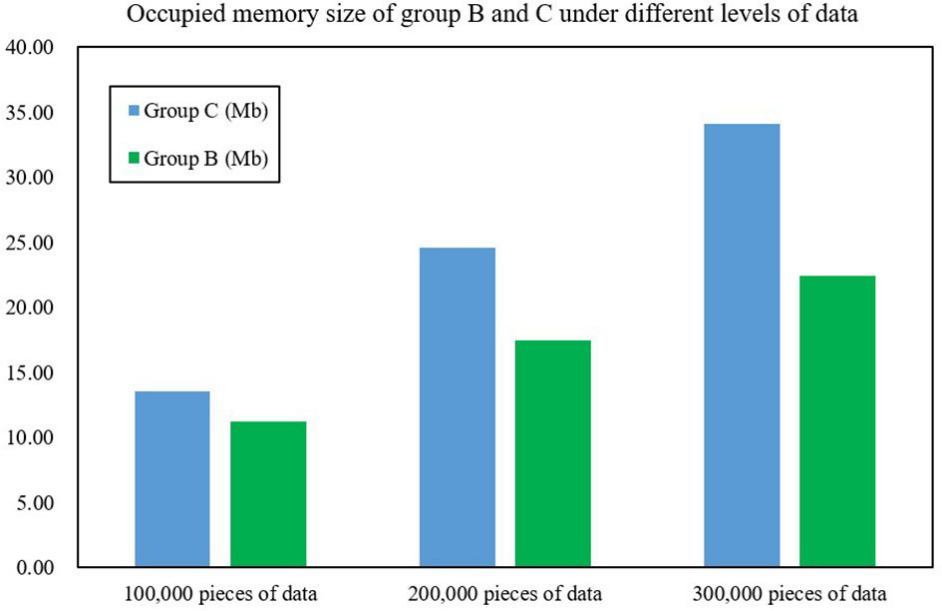
Occupied memory size of groups B and C under different levels of data.

In order to further evaluate the performance of the FAST-join algorithm, this article also introduces the Spark component optimized by the Equi-join algorithm (Haoqiong et al., 2014) (another large table equivalent join optimization algorithm), and deploys it separately as cluster C under the same configuration. It also compares the processing time and memory size of the cluster for 100,000, 200,000 and 300,000 pieces of data. Figures 8, 9 show the processing time and memory size of clusters A and C, respectively. After experimental testing, the average data processing time of Group B was only 59.37% of that of Group C, an average reduction of 68.74% compared with Group C. The average occupied memory size of Group B only accounts for Group A's 73.23%, an average reduction of 37.80% compared to Group C. This shows that the processing time and occupied memory size of Spark optimized by the FAST-join algorithm is significantly reduced compared with the Equi-join algorithm.

## 4 Conclusions

In this study, a data processing method for farmland environmental monitoring based on improved Spark components was designed. The FAST-join algorithm is introduced to optimize Spark's association query (shuffle processing capacity) and cluster computing. After experimental testing, the results showed that the improved Spark has an excellent performance in data table query and cluster computing and can efficiently process farmland environmental monitoring data, which can effectively improve the efficiency of inter-data table querying and cluster computing.

We envisage applying this farmland data processing method to more data types, such as images of farmland pests and diseases, videos, and data from various intelligent operating equipment. These data types are quite different from farmland environmental monitoring data, such as images and video, and the amount of data is relatively large, which requires the introduction of Spark's compression mechanism to compress these data and the use of corresponding algorithms to convert unstructured data into structured data, to realize real-time and rapid monitoring of farmland by the cloud platform of pest and disease situation. Through these follow-up in-depth studies, the scope of application of this data processing method will be expanded, and the applicability of this method in farmland monitoring will be improved.

## Data availability statement

The original contributions presented in the study are included in the article/supplementary material, further inquiries can be directed to the corresponding author.

## Author contributions

RT: Conceptualization, Data curation, Formal analysis, Investigation, Methodology, Software, Validation, Visualization, Writing—original draft, Writing—review & editing. NA: Data curation, Writing—review & editing, Funding acquisition, Project administration, Resources, Supervision. MT: Data curation, Project administration, Resources, Supervision, Validation, Writing—review & editing.
